# Realigning global health governance: WHO at a crossroads

**DOI:** 10.1186/s12939-020-01297-y

**Published:** 2020-10-23

**Authors:** Irene Torres, Osvaldo Artaza, Barbara Profeta, Cristina Alonso, JaHyun Kang

**Affiliations:** 1 Fundacion Octaedro, El Zurriago E8-28 y Ave. De los Shyris, Quito, Ecuador; 2 Faculty of Health Sciences, University of the Americas, Santiago de Chile, Chile; 3 Independent consultant, Fribourg, Switzerland; 4 Harvard T. H. Chan School of Public Health, Boston, MA USA; 5 College of Nursing and Research Institute of Nursing Science, Seoul National University, Seoul, Korea

It has been over 5 years since the Ebola epidemic, meaning the World Health Organization (WHO) had time to improve coordination, distribution of responsibilities and effective resource mobilization for a rapid response to a pandemic [1, 2]. Reforms would have addressed demonstrated failings and entrenched weaknesses that enabled the Ebola virus to rapidly spread; instead, WHO’s role has been openly questioned, with geopolitics holding center stage over concern for global population health [3].

While recommendations for reform give the illusion of progress, reminders of the unmet conditions for success are constant. The private sector is considered a crucial partner in an effective health crisis response such as the unfolding COVID-19 pandemic [1, 4]. Nevertheless, international trade agreements are weighted more heavily than international health regulations. As an example, legally binding instruments such as the Framework Convention on Tobacco Control [5] were approved decades after scientific consensus on the urgent need to protect public wellbeing.

This influence is clear in health research, where instead of prioritizing disease prevention, the focus remains on cures [6], which provide higher returns on investment. More importantly, little has been said about the impossibility for WHO to truly defend global public health when forced to broker between private interests, and political influence from large economies driven by transnational corporations, and the protection of the most vulnerable.

With WHO acting only partially as a lead authority, it is unfair to place blame on the sole organization’s shortcomings, be they financial, institutional or informational, for the glaring flaws in the COVID-19 response. Some rich member states equally failed to protect their populations, ignoring warnings on risk preparedness [7] to safeguard economic gains. The pandemic rendered patent not only the absence of an effective global health governance system, but possibly also the very existence of a global space understood as the sum of sovereign territories.

The post-war world in which the United Nations were born and developed has drastically changed, consolidating widespread inequities while creating new complexities, which were further propelled by the COVID-19 pandemic. Digital technologies have reshaped the contours of ownership and accountability, largely to the benefit of high-income countries and multinational companies, thereby calling for a renegotiated commitment.

The imperative of global health governance requires realigning international cooperation so that WHO, once again at a crossroads, can unequivocally prioritize and protect the common good. This is the only way to avoid replicating the current global catastrophe in the future.

## Data Availability

Not applicable.
